# Inhibition of Inflammatory Gene Expression in Keratinocytes Using a Composition Containing Carnitine, Thioctic Acid and Saw Palmetto Extract

**DOI:** 10.1093/ecam/nep102

**Published:** 2011-06-08

**Authors:** Sridar Chittur, Brian Parr, Geno Marcovici

**Affiliations:** ^1^State University of New York (SUNY), Albany, NY, USA; ^2^Advanced Restoration Technologies, Inc., 9035 North 15th Place, Phoenix, AZ 85020, USA

## Abstract

Chronic inflammation of the hair follicle (HF) is considered a contributing factor in the pathogenesis of androgenetic alopecia (AGA). Previously, we clinically tested liposterolic extract of *Serenoa repens* (LSESr) and its glycoside, **β**-sitosterol, in subjects with AGA and showed a highly positive response to treatment. In this study, we sought to determine whether blockade of inflammation using a composition containing LSESr as well as two anti-inflammatory agents (carnitine and thioctic acid) could alter the expression of molecular markers of inflammation in a well-established *in vitro* system. Using a well-validated assay representative of HF keratinocytes, specifically, stimulation of cultured human keratinocyte cells *in vitro*, we measured changes in gene expression of a spectrum of well-known inflammatory markers. Lipopolysaccharide (LPS) provided an inflammatory stimulus. In particular, we found that the composition effectively suppressed LPS-activated gene expression of chemokines, including *CCL17*, *CXCL6* and *LTB(4)* associated with pathways involved in inflammation and apoptosis. Our data support the hypothesis that the test compound exhibits anti-inflammatory characteristics in a well-established *in vitro* assay representing HF keratinocyte gene expression. These findings suggest that 5-alpha reductase inhibitors combined with blockade of inflammatory processes could represent a novel two-pronged approach in the treatment of AGA with improved efficacy over current modalities.

## 1. Introduction

The pathogeneses of benign prostatic hyperplasia (BPH) and androgenetic alopecia (AGA) are mediated in part by the transcriptional pathways downstream of the steroid hormone androgen receptor (AR). The predominant ligand in these tissues is dihydrotestosterone (DHT), which is formed by the conversion of the inactive form of testosterone (T) and is catalyzed by the enzyme 5-alpha reductase (5-AR).

Anti-androgens and inhibitors of 5-AR have proven effective in the treatment of BPH as well as AGA, attesting to their common disease mechanisms. Both the pharmaceutical compound, finasteride (Proscar or Propecia) and the liposterolic extract of *Serenoa repens* (LSESr) have shown efficacy in the treatment of BPH and AGA. Notably, in a direct comparison of LSESr against finasteride, it has been reported that LSESr exhibited a 3-fold greater inhibition of 5-AR in *in vitro* assays [1].

Finasteride (at a dose of 5 mg, as in Proscar) is used as the treatment of choice for BPH, particularly in the USA. A number of well-controlled studies point to its efficacy in ameliorating the signs and symptoms of BPH [2]. In large, double-blind, placebo-controlled clinical studies recruiting over 1600 patients, it was shown that the administration of finasteride reduced the size of the prostate by a mean of 22%, following 6 months of therapy [3]. Likewise, multiple well-controlled clinical trials reinforce the utility of LSESr in the setting of BPH, predominantly in Europe [4]. Investigators have found that LSESr is well tolerated and has greater efficacy than placebo and similar efficacy to finasteride in improving symptoms in men with BPH [5].

During the course of a clinical trial of Proscar for BPH, it was noted serendipitously that there was a cessation of hair loss in study subjects receiving drug [6]. Therefore, finasteride (at a dose of 1 mg; as in Propecia) was subsequently investigated in clinical trials for the treatment of men with AGA. In affected individuals, long-term treatment with finasteride 1 mg/day over 5 years was well-tolerated, led to visible improvements in scalp hair growth and slowed the further progression of hair loss that occurred without treatment [7]. LSESr is well known for its role in BPH as a 5-AR inhibitor, leading us to postulate a similar effect in AGA.

Previously, we tested LSESr and its glycoside, *β*-sitosterol, in subjects with AGA and showed a highly positive response to treatment. The blinded investigative staff assessment reported that 60% of study subjects dosed with the active study formulation were rated as improved at the conclusion of the trial and established the effectiveness of naturally occurring 5-AR inhibitors against AGA for the first time [8].

Notwithstanding, the common mechanism of androgens in their pathogenesis, several lines of emerging evidence suggest that both BPH and AGA are also associated with significant dysregulation in the expression of inflammatory cytokines [9]. For example, gene expression profiling of prostate tissue from BPH patients revealed molecular signatures containing genes associated with inflammation [10]. Likewise, chronic inflammation of the hair follicle (HF) is also considered a contributing factor in AGA [11]. Another recent study reported a relationship between moderate to extensive alopecia and chronic low-grade inflammation [12]. Histologically, it has been shown that in scalp biopsies from AGA patients, sustained follicular inflammation with connective tissue remodeling eventually results in permanent hair loss and, thus, is described as a possible cofactor in the complex etiology of the disorder [11].

On the basis of such findings, the utility of treating BPH-affected patients with anti-inflammatory agents in combination with 5-AR inhibitors is currently under investigation. In one study, combination therapy with alpha(1)-adrenergic receptor antagonists [alpha(1)-ARAs] and the 5-AR inhibitor finasteride was significantly more effective than either component alone in reducing BPH-related symptoms (*P* = .006 versus doxazosin monotherapy; *P* < .001 versus finasteride monotherapy) and lowering the rate of overall clinical progression (*P* < .001 versus either monotherapy alone) [13]. The greatest efficacy was observed in patients with a markedly enlarged prostate, more severe symptoms and higher PSA levels. These data suggest that the treatment of BPH is enhanced by the use of anti-inflammatory agents in combination with 5-AR blockade [14]. Taken together, these lines of evidence led us to postulate that inflammation is a rational therapeutic target in pattern hair loss (AGA). In this study, we sought to determine whether blockade of inflammation using a composition containing LSESr, carnitine and thioctic acid (TA) could alter the expression of molecular markers of inflammation in a well-established *in vitro* system. We found that our compound effectively repressed LPS-activated expression of a number of genes involved in skin inflammation and apoptosis. Our findings suggest that 5-AR inhibitors combined with blockade of inflammatory processes could represent a novel two-pronged approach in the treatment of AGA with improved efficacy over current modalities.

## 2. Methods

### 2.1. Cell Culture and Challenge

Human keratinocyte cells (HaCaT) were grown in low glucose DMEM supplemented with 10% FBS, 2 mM l-glutamine, 1 mM sodium pyruvate solution, 0.1 mM NEAA, 100 U ml^−1^ penicillin, 100 *μ*g ml^−1^ streptomycin at 37°C and 5% CO_2_. They were subcultured on Day 2 and grown to 80% confluency on six-well plates.

The HaCaT cells were treated with either 100 ng or 200 ng of LPS (Sigma, St Louis, MO, USA) for 2 h. The media was then replaced with fresh LPS at the same concentration along with the LSESr, TA and carnitine test substance, hereafter designated as TS-050508A (1 : 1000). Each condition was carried out in triplicate. The cells were harvested in 1 ml trizol after 22 h. Control wells were treated with 100 ng or 200 ng for 24 h and harvested in 1 ml trizol. Alternately, the HaCat cells were treated with TS-050508A (1 : 1000) for 2 h. The media was then replaced with either 100 ng or 200 ng of LPS along with fresh TS-050508A (1 : 1000). Each condition was carried out in triplicate. The cells were harvested in 1 ml trizol after 22 h. Control wells were treated with TS-050508A (1 : 1000) for 24 h and harvested in 1 ml trizol. Cell viability was determined by trypan blue staining.

### 2.2. Trypan Blue Staining

The HaCaT cells were treated with 0.5 ml 0.05% trypsin–EDTA and placed at 37°C in 5% CO_2_ for 12 min. The cells were neutralized with 0.5 ml Dulbecco's Modified Eagle's Medium (DMEM) 10% Fetal Bovine Serum (FBS). 30 *μ*l of trypan blue was mixed with 30 *μ*l of cell suspension. Approximately 10 *μ*l was loaded into a haemocytometer and the cells were counted. Counts were taken at 3, 6, 12 and 24 h for the No LPS, 100, 200 and 400 ng LPS conditions, respectively. Under our culture conditions and over this concentration range of LPS, the effects of LPS on cell viability, as measured by trypan blue, were variable but there was no consistent decrease in viability as LPS concentration increased. Therefore, we used LPS at the lower end of this concentration range to ensure cell viability. Our findings are consistent with those observed by others [15].

### 2.3. Nucleic Acids Preparation and PCR-Based Expression Array

Total RNA was isolated from all samples using standard protocols and further cleaned using RNeasy mini columns according to the manufacturer's recommendations (Qiagen, Gaithersburg, MD, USA). The RNA was deemed of good quality for the 260/280, 260/230 and the 28s/18s ribosomal ratios were close to 2. The RNA (500 ng) was then reverse transcribed to cDNA using the protocols as suggested in the RT^2^ First strand kit (SA Biosciences, Frederick, MD, USA). The cDNA was then used to prepare a master mix as per the RT^2^ qPCR kit protocol (SA Biosciences). A 10 *μ*l aliquot of this mix was loaded on to the wells in a custom 384-well plate (designed to include most of the genes in the inflammation and cytokine response pathway). The cycle time (C_t_) values from these reactions were analyzed using the PCRarray data analysis template to calculate the ΔΔCt and the fold change [fold change = 2^(−ΔΔCt)^].

### 2.4. RT–PCR Validation of Results

Equal amount of RNA (1 *μ*g) was taken for all samples and reverse transcription was performed using RT^2^ First Strand kit (Cat# C-03) from SA Biosciences. The total volume of the reaction was 20 *μ*l, and the final RT product was diluted to a volume of 100 *μ*l. Individual PCR assays were performed with the off-the-shelf pre-validated primer pairs from SA Biosciences on the BioRad iCycler iQ using RT^2^ SYBR Green/ROX qPCR Master Mix (Cat# PA-011, SA Biosciences). PCR assays for each gene target were performed in triplicates for each sample. Each PCR reaction contained cDNA synthesized from 10 ng total RNA. Relative changes in gene expression were calculated using the ΔΔC_t_ (threshold cycle) method. Fold change values are calculated using the formula 2^−ΔΔCt^. There were three biological replicates in each treatment group. Comparisons between groups were made using unpaired Student's *t*-tests with the level of significance set at *P* < .05.

## 3. Results

### 3.1. Test Compound Suppressed LPS-activated Gene Expression

In this study, we used a well-validated *in vitro* assay representative of HF keratinocytes, specifically, stimulation of cultured HaCaT cells. We measured changes in gene expression of a spectrum of well-known inflammatory markers, using lipopolysaccharide (LPS) as an inflammatory stimulus. Using untreated cells as a baseline for gene expression, we tested our compound under seven conditions: (i) 100 ng LPS alone (low dose); (ii) 200 ng LPS alone (high dose); (iii) compound alone (1 : 1000); (iv) compound followed by low-dose LPS; (v) compound followed by high-dose LPS; (vi) low-dose LPS followed by the compound and lastly (vii) high-dose LPS followed by the compound. Overall, we found that treating HaCaT cell cultures with our compound resulted in reduced LPS-mediated inflammatory gene expression without inducing a negative effect on cell viability. In particular, we found that the composition effectively suppressed LPS-activated gene expression of chemokines associated with pathways involved in inflammation and apoptosis.

### 3.2. Global Expression Analysis Reveals Inhibition of Inflammatory Pathways

The RT^2^ Profiler PCR Array from SuperArray was used to interrogate the effect of the novel composition on inflammation. This array contains 384 well-validated genes known to be involved in stromal, endothelial and epithelial inflammatory pathways. The classes of genes represented on the array included the CC ligand chemokines, toll-like receptor genes and their ligands, epithelial cell adhesion molecules, and members of the CXC chemokine family and leukotriene lipid mediators derived from the 5-lipoxygenase pathway of arachidonic acid metabolism.

**Figure 1:**
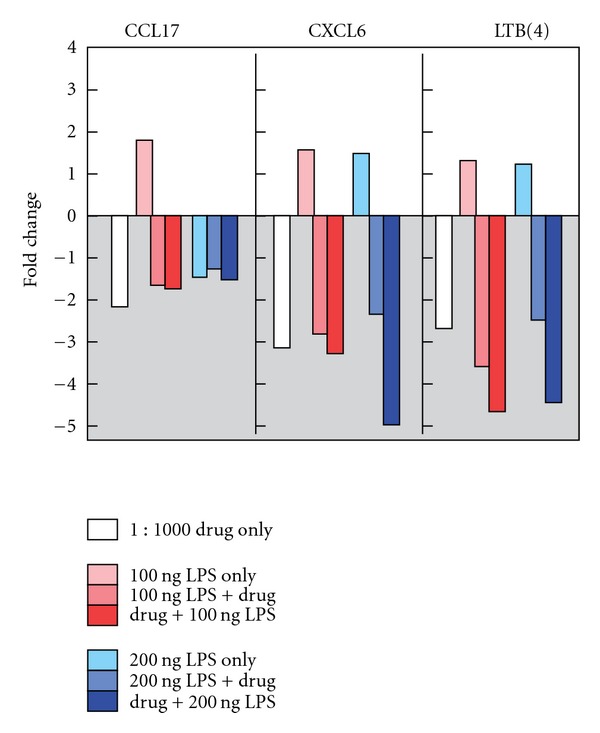
Noteworthy fold change occurring in CCL17, CXCL6 and LTB(4).

When measured against the untreated cells, we noted statistically significant (*P* ≤ .05) fold changes in the following genes: *CCL3*, *CCL8*, *CCL17*, *CCL24*, *CCR7*, *CXCL2*, *CXCL6*, *IL18RAP*, *IL1A*, *IL1B*, *IL1F10 LTB(4)* and *TLR4*, (Table 1). Below we will focus on three genes, *CCL17*, *CXCL6* and *LTB(4)* (Figure 1).

### 3.3. Chemokine Ligand CCL17

In this study, our compound showed modest but statistically significant anti-inflammatory activity against CCL17. The HaCaT cells treated with 100 ng LPS displayed a 1.76 up-regulated fold change as compared to untreated controls. Dosing the cells with 200 ng LPS resulted in a down-regulation of CCL17 by a factor of −1.43-fold. The cells treated with the test compound alone down-regulated expression of CCL17 by a factor of −2.17 from baseline, whereas cells treated with compound followed by low-dose LPS resulted in a down-regulation of −1.71-fold. Overall, the results showed that our compound down-regulated the inflammatory marker CCL17. The test composition also blocked the up-regulation of message, both prior to and subsequent to LPS-mediated challenge.

### 3.4. CXC Chemokine Ligand, CXCL6

We show statistically significant anti-inflammatory activity in an LPS-mediated model of skin inflammation via the marker CXCL6. The low-dose LPS-only (100 ng) stimulated cells resulted in CXCL6 being up-regulated by 1.57-fold. When measured against baseline, the test compound alone down-regulated expression of CXCL6 by a factor of −3.09. After incubating the cells with the test compound followed by exposure to low-dose LPS, CXCL6 expression showed a −3.28-fold change as measured against baseline. These results show a blockade of CXCL6 inflammatory message in the LPS-stimulated HaCaT cells by the test compound.

### 3.5. Leukotriene B(4)

In this study, our test compound showed a noteworthy effect in the leukotriene [LTB(4)] marker. The HaCaT cells treated with 100 ng LPS inflammatory agonist displayed an up-regulated fold change for LTB(4) of 1.33 when measured against baseline. Gene expression of LTB(4) showed a down-regulation of −2.64-fold in the cells treated with the test compound alone. A 100 ng LPS incubated cells followed by the test compound showed marked change in LTB(4) expression demonstrating a −4.59-fold change as measured against baseline. These results show that our compound blocked the LPS-stimulated up-regulation of the inflammatory marker LTB(4).

In summary, we found that our test compound attenuated expression for markers of inflammation, and specifically for CCL17, CXCL6 and LTB(4). Our data support the hypothesis that the test compound exhibits anti-inflammatory characteristics in a well-established *in vitro* assay representing HF keratinocyte gene expression.

## 4. Discussion

In this study, we sought to demonstrate that a test compound containing LSESr, carnitine and TA represses LPS-activated expression of inflammatory genes using a well-validated *in vitro* assay representative of HF keratinocytes, specifically, the LPS-stimulated HaCaT cells [16, 17]. We assayed for changes in gene expression across a spectrum of well-characterized inflammatory markers and found that our test compound demonstrates anti-inflammatory properties *in vitro*. Plant extracts or mixtures comprise a repertoire of chemical entities that have pleiotropic effects on cellular physiology. As such, they have great potential in the multi-target approach to diseases [18, 19]. Microarray analysis of gene expression following exposure of cells to plant extracts can be useful for elucidating the molecular networks impacted by herbal extracts and mixtures [20]. In particular, plant-based therapies have proven efficacious in the treatment of inflammatory skin disorders, such as atopic dermatitis [21].

Here, we found that treating HaCaT cell cultures with our compound resulted in a statistically significant (*P* < .05) reduction of LPS-mediated inflammatory gene expression without inducing a negative effect on cell viability. Specifically, we noted that the composition effectively suppressed LPS-activated gene expression of chemokines CCL17, CXCL6 and LTB(4). The over-expression of each of these markers has previously been observed in the setting of inflammation and apoptosis. We focused here on short term and immediate changes in gene expression, using array hybridization, followed by qPCR. The gene expression fold changes we observed were statistically significant. Our approach is supported in the literature by many other examples using the same strategy, in studying acute responses to stimuli such as LPS, in addition to UV irradiation and wounding in the HaCaT cells and keratinocytes [22–25].

Our rationale for selecting CXCL6, CCL17 and LTB(4) for further study relates to their role in inflammatory disorders of the skin, mucosae and epithelia. CXCL6 is a CXC chemokine expressed by macrophages, epithelial and mesenchymal cells during inflammation. Recently, it was shown that the transcriptome of the aging prostate stroma is characterized by the up-regulation of several genes that encode secreted inflammatory mediators, including CXCL6 [26]. Similarly, in the skin, CCL17 (a CC chemokine ligand) is up-regulated under stress, injury or inflammation [27]. CCL17 is also over-expressed in stromal, endothelial and epithelial tissues in autoimmune disorders including the inflammatory bowel diseases, Crohn's disease and ulcerative colitis (UC) [28]. Finally, LTB(4) is a lipid inflammatory mediator derived from membrane phospholipids by the sequential actions of cytosolic phospholipase A2 (PLA2), 5-lipoxygenase (5-LO) and leukotriene A(4) [LTA(4)] hydrolase. LTB(4), best known as a neutrophil chemoattractant, is now recognized to exert other important effects contributing to inflammatory and immune responses. Reinforcing the connection between androgen-mediated disorders and inflammatory processes, LTB(4) expression has been demonstrated in acne vulgaris [29].

While LPS caused upregulation of CXCL6 and LTB(4) at both doses as expected, we observed a modest downregulation of CCL17 at the higher dose of LPS. Likewise, in a recent study using the LPS challenge cell culture model, the authors noted that their responses might have also differed from predicted expectation based on the presence or absence of specific cell-surface receptors in 2D culture versus the 3D environment [30]. Thus, the observation that high-dose LPS itself partially lowers CCL17 expression does not change our interpretation, since the treatment represses expression beyond the level of high-dose LPS. The overall result of our experiment is that LPS challenge (at low dose) induces the expected increase in CCL17, and the treatment group demonstrates potent downregulation of CCL17.

**Figure 2:**
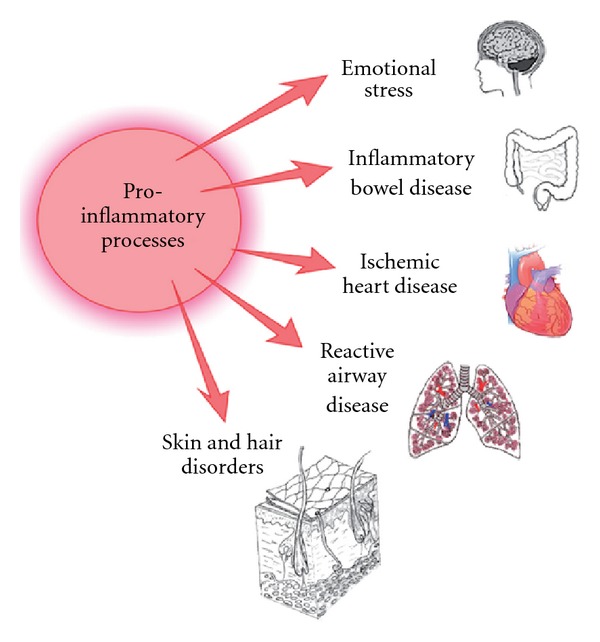
Many organ systems are affected by pro-inflammatory processes.

A large body of evidence supports the involvement of pro-inflammatory processes in the development and progression of numerous disorders, including inflammatory bowel disease, ischemic heart disease and asthma (Figure 2) [18]. Chronic inflammation is recognized at the molecular and cellular levels as the final common pathway of many systemic and degenerative diseases, including those affecting the skin [31]. It has also been observed that inflammation, even that induced by emotional stress, plays a role in hair loss, and recent studies point to inflammation as a contributing factor to AGA [32]. In a murine model, the authors demonstrated that psychoemotional stress indeed alters actual HF cycling *in vivo*, that is, prematurely terminates the normal duration of active hair growth (anagen) [33]. Furthermore, inflammatory events deleterious to the HF are present in the HF environment of stressed mice (perifollicular macrophage cluster, excessive mast cell activation). It has also been shown experimentally that high-dose pro-inflammatory cytokines induce apoptosis of HF keratinocytes *in vivo* [34].

Histologically, sustained microscopic follicular inflammation with connective tissue remodeling is a noted cofactor in the complex etiology of AGA [35]. Disruption of HF homeostasis, via injury, insult or inflammation results in a shift from proliferation to apoptosis. Cumulatively, these findings underline the susceptibility HFs display when subjected to inflammatory insult.

In order to clarify the rationale leading to this study, l-carnitine, TA and LSESr operate through distinct, but potentially interrelated, biochemical and molecular mechanisms, with each representing an important component in the design of the test formula. Carnitine, a potent anti-inflammatory agent, biosynthesized from the amino acids lysine and methionine is required for the transport of fatty acids from the cytosol into the mitochondria during the breakdown of lipids for the generation of metabolic energy [36]. Carnitine exists in two stereoisomers: its biologically active form is l-carnitine, while its enantiomer, d-carnitine, is biologically inactive [37].

Accumulating evidence suggests that l-carnitine may play a significant role in prevention and treatment of numerous diseases as well as protection from accelerated aging that result from oxygen free-radical damage, inflammation and glycation (non-enzymatic glycosylation). Documented benefits from l-carnitine include clinical improvement in patients diagnosed with diabetes, cardiovascular disease, hypertension, congestive heart failure, age-related deterioration of brain function and vision and immune function [38]. One line of research suggests that carnitine may possess the ability to promote hair growth *in vitro* by increasing energy supply to the rapidly prolife rating and the energy-consuming anagen hair matrix [39].

The naturally occurring antioxidant TA was first described as an essential cofactor for the conversion of pyruvate to acetyl-CoA, a critical step in respiration [40]. TA is now recognized as a compound with many biological functions, such as the modulation of pathogenic inflammatory events, including those in the skin [41].

In addition to the two constituents described above, the test compound also contains the LSESr derived from the fruit of saw palmetto. LSESr is highly enriched with fatty acids and phytosterols and has been used historically to treat urinary tract symptoms, including BPH [42]. LSESr has been shown to block both isoforms of 5-alpha reductase (types I and II) with the added benefit that, in contrast to finasteride, LSESr does not interfere with PSA (prostate-specific antigen) levels [43]. As previously noted, we were the first to clinically demonstrate the utility of LSESr against AGA [8].

While the genetic architecture of AGA has not yet been determined, it is clearly a polygenic disorder in which multiple genes, hormonal pathways and environmental factors contribute to phenotype. To date, DHT modulation via 5-AR blockade remains the only druggable target shown to ameliorate the progression of AGA [44]. An important feature of our hypothesis hinges on the enhanced efficacy of combining anti-inflammatory agents with 5-AR inhibitors.

A paradigm for enhanced performance via concomitant therapy already exists in the treatment of skin disease. For example, studies show that the addition of a topical corticosteroid to imidazole therapy increases the bioavailability and prolongs the activity of the antimycotic, while rapidly reducing inflammatory symptoms [45].

In several studies, a synergy between TA and vitamin E has been described, and potent antioxidant effects can be obtained when both antioxidants are simultaneously used. Specifically, recent findings showed that the combination of TA plus vitamin E effectively reduces oxidative damage in brain and cardiac ischemia as well as in other pathological events associated with tissue inflammation and secondary to the formation of reactive oxygen species (ROS) [46]. In *in vitro* and animal studies, l-carnitine, particularly when used in combination with TA, has been shown to reverse age-related changes in numerous tissues, systems and pathways [47].

Since the clinical success rate for treatment of AGA with androgen blockade is limited, there is enormous unmet medical need for patients who are refractory to current therapy. Recognizing that inflammation contributes to a wide range of diseases, including those affecting the skin and hair, it is our hypothesis that the blockade of inflammation represents a new and potentially viable therapeutic avenue. We suggest that our data may offer new insight into the potential for combining anti-inflammatory compounds with 5-AR inhibitors for the treatment of AGA.

## Figures and Tables

**Table 1:** Gene expression profile of inflammatory markers expressing statistically significant fold change.

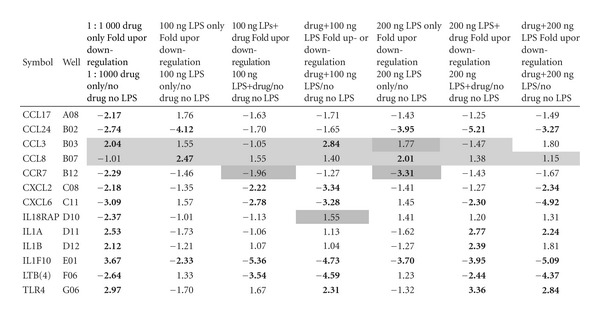

Values that have been highlighted showed weak amplification in qPCH; Light gray shadings indicates both control and test samples showed weak amplification; Dark gray shadings indicates only test sample shows weak amplification. Bold text indicates stastically significant fold changes.
